# Developing community-driven quality improvement initiatives to enhance chronic disease care in Indigenous communities in Canada: the FORGE AHEAD program protocol

**DOI:** 10.1186/s12961-016-0127-y

**Published:** 2016-07-26

**Authors:** Mariam Naqshbandi Hayward, Jann Paquette-Warren, Stewart B. Harris, On behalf of the FORGE AHEAD Program Team

**Affiliations:** Centre for Studies in Family Medicine, Western Centre for Public Health and Family Medicine, Department of Family Medicine, Schulich School of Medicine and Dentistry, Western University, 1511 Richmond Street, London, Ontario N6K 3K7 Canada

**Keywords:** Indigenous, First Nations, Chronic disease, Diabetes, Quality improvement, Complex interventions

## Abstract

**Background:**

Given the dramatic rise and impact of chronic diseases and gaps in care in Indigenous peoples in Canada, a shift from the dominant episodic and responsive healthcare model most common in First Nations communities to one that places emphasis on proactive prevention and chronic disease management is urgently needed.

**Methods:**

The Transformation of Indigenous Primary Healthcare Delivery (FORGE AHEAD) Program partners with 11 First Nations communities across six provinces in Canada to develop and evaluate community-driven quality improvement (QI) initiatives to enhance chronic disease care. FORGE AHEAD is a 5-year research program (2013–2017) that utilizes a pre-post mixed-methods observational design rooted in participatory research principles to work with communities in developing culturally relevant innovations and improved access to available services. This intensive program incorporates a series of 10 inter-related and progressive program activities designed to foster community-driven initiatives with type 2 diabetes mellitus as the action disease. Preparatory activities include a national community profile survey, best practice and policy literature review, and readiness tool development. Community-level intervention activities include community and clinical readiness consultations, development of a diabetes registry and surveillance system, and QI activities. With a focus on capacity building, all community-level activities are driven by trained community members who champion QI initiatives in their community. Program wrap-up activities include readiness tool validation, cost-analysis and process evaluation. In collaboration with Health Canada and the Aboriginal Diabetes Initiative, scale-up toolkits will be developed in order to build on lessons-learned, tools and methods, and to fuel sustainability and spread of successful innovations.

**Discussion:**

The outcomes of this research program, its related cost and the subsequent policy recommendations, will have the potential to significantly affect future policy decisions pertaining to chronic disease care in First Nations communities in Canada.

**Trial registration:**

Current ClinicalTrial.gov protocol ID NCT02234973. Date of Registration: July 30, 2014.

**Electronic supplementary material:**

The online version of this article (doi:10.1186/s12961-016-0127-y) contains supplementary material, which is available to authorized users.

## Background

Chronic diseases are considered to be among the most preventable of all health problems, yet WHO predicts that, in 2030, chronic diseases will be the seventh leading global cause of death [1]. The chronic nature of these diseases has substantial cost implications and has generated a priority need to modify healthcare systems to improve the effectiveness of care delivery [2–4]. Among the most common chronic diseases, type 2 diabetes mellitus (T2DM) affects an increasing number of people worldwide [5, 6]. Of great concern is the fact that Indigenous peoples experience significantly worse health outcomes associated with T2DM, with prevalence rates that are 2–5 times higher than the general population [7–9]. A recent national study that assessed the T2DM burden and clinical care gaps among 19 First Nations communities found a much higher disease burden in a relatively younger population with significant care gaps compared to the general population [8]. Two-thirds of the study participants had chronic kidney disease [7], 13% had coronary artery disease, and just over 10% were diagnosed with neuropathy and retinopathy [8]. The mortality rate due to diabetes for Indigenous peoples living in Canada is also higher, at 19.5 per 100,000 people compared to the general population rate of 13.3 per 100,000 [10]. The utilization of physicians, hospitals and dialysis has been reported to be 40–60% higher among First Nations compared to the general population with T2DM in Saskatchewan, leading to higher healthcare costs [11].

The higher rates of adverse health outcomes in Indigenous peoples are related to an array of factors, including the social determinants of health (i.e. low income, lack of education, high unemployment, poor living conditions, lack of social support, negative stereotyping and stigmatization) [12], lifestyle (diet and physical activity), genetic susceptibility, and historic-political and psycho-social factors stemming from a history of colonization that severely undermined Indigenous values, culture and spiritual practices [13]. Barriers to care that are unique to First Nations communities also exacerbate the problem with fragmented healthcare, poor chronic disease management, high healthcare staff turnover and limited, or non-existent, surveillance of chronic diseases [12].

The Federal Government’s role in the provision of health services to registered First Nations living on-reserve and to Inuit living in their traditional territories, is primarily through the public health and prevention services offered by the First Nations and Inuit Health Branch [14]. Home and community care is provided in over 650 First Nations and Inuit communities. The Aboriginal Diabetes Initiative supports health promotion and T2DM prevention activities and services delivered by trained community diabetes workers and health service providers [15]. Additionally, the First Nations and Inuit Health Branch provides non-insured health benefits, such as prescription drugs, dental and vision coverage, to all registered First Nations and Inuit. As for physician and hospital care, these services are provided by provincial and territorial governments [16] and, depending on the region and degree of isolation of the community, the model of service ranges from nursing stations supported by fly-in physicians and nurses to fully-staffed community hospitals [15]. Furthermore, higher rates of adverse health outcomes are impacted by the focus of primary care services on acute and episodic care management rather than chronic disease care [17] and, in remote and isolated communities, the lack of physician support exacerbates the reliance on an acute care approach because nurses and other non-physician health professionals are overburdened [18]. This, combined with often poor coordination between provincially funded hospitals/specialty care and federal nursing care in most First Nations communities [19], has resulted in significantly higher admissions to hospitals for ambulatory care conditions [20–22].

Sustained improvements in the quality of care of chronic diseases has been achieved in the United States, Australia and Canada through the Indian Health Services national T2DM surveillance and audit/feedback programs, the Audit and Best Practice for Chronic Disease program, and the DREAM3 study, respectively [23–25]. The Indian Health Services in the United States implements the Diabetes Care and Outcomes Audit, an annual nationwide voluntary self-audit of T2DM care and outcomes. Since the program’s inception in 1996, blood glucose control has steadily improved from a mean glycosylated haemoglobin (HbA1c) of 9.0% to 8.0% in 2011 [24]. Low density lipoprotein cholesterol values have been lowered by 20%, thus reducing the risk of cardiovascular disease [24]. The Audit and Best Practice for Chronic Disease program in Australia used continuous quality improvement (QI) and action research approaches (pre-post evaluation) with eight community health centres that were supported to identify their own goals to improve chronic disease care (including T2DM), develop strategies to achieve these goals, and to assess the effectiveness of their strategies. Through two annual cycles of continuous QI, the Audit and Best Practice for Chronic Disease study found an improvement in HbA1c testing from 41% to 72%, and an increase in the proportion of people from 19% to 28% with an HbA1c at target (<7.0%) [23]. The DREAM3 study in Canada, a randomized trial using patients with diabetes in a Saskatchewan First Nations community, demonstrated a significant reduction of blood pressure within 1 year, which was sustained during a 2-year self-maintenance period [25].

These programs include various aspects of the most recognized approach to optimize chronic disease care: the Chronic Care Model (CCM) [26] and the Expanded Chronic Care Model (ECCM) [27]. The CCM outlines ways in which healthcare partners can work together to improve care along its entire continuum by stressing the importance of key components, including patients, their families, the community and the healthcare system [28]. The ECCM adds elements of population health including determinants of health [27]. Provincial and federal governments in Canada have embraced these models [29, 30]. QI interventions using the CCM have demonstrated improvement to T2DM outcomes [31–33]. The recently published Dulce intervention [33] included a patient registry, surveillance, trained nurses following a management protocol, and trained peer coaches. In a controlled evaluation, a clinical and cost-effective result was found (HbA1c: 8.3% vs. 10.4% at 1 year). Meta-analyses of QI initiatives have identified successful components [34, 35]. Tricco et al. [35] found the most effective strategies in ameliorating HbA1c control included targeting healthcare systems (team changes and case management) and patients (self-management promotion and education). For patients with poor glycaemic control (HbA1c ≥ 8%), the most effective strategies were focused on team changes and case management. As for critical elements to include in programs that aim to improve chronic care, a combination of off-site learning/classroom sessions, practice-based information-technology support, and practice coaching that provides dedicated time to learn how to improve chronic care, as well as team-building within and across teams, hands-on information-technology training, and flexibility to meet individual practice needs have been reported as the most effective [36].

The literature on primary healthcare disparities and QI interventions in Indigenous populations consistently notes that participatory research principles and cultural tailoring and competencies need to be priorities [37, 38]. All three concepts stress the co-construction of initiatives between researchers and the people affected by the issues under study (e.g. patients, community members, community health professionals, representatives of community-based organizations) and/or decision-makers who apply research findings (e.g. community leaders, health managers, policymakers). For health intervention research, participatory research strengthens relations between the community and academia, ensures the relevancy of research questions, increases the capacity of data collection, analysis and interpretation, reduces the ‘iatrogenic’ effects of research, and enhances program recruitment, sustainability and extension beyond funding [39, 40]. Further, participatory research increases communities’ capacity to identify and solve their [41, 42] and the decision-makers’ and service providers’ ability to mobilize resources, improve policies and enhance professional practices [43, 44]. Cultural leverage, a process incorporating cultural competency and participatory research principles to develop and implement interventions aimed at reducing health disparities, has shown the most success with culturally specific patient navigators and community health workers [38].

In light of the fact that Canada is spending an increasing share of its revenue on healthcare yet falling behind other industrialized nations in obtaining value for its investment related to performance on safety, quality, equity and efficiency [45, 46], the Transformative Community-based Primary Healthcare initiative was launched by the Canadian Institutes of Health Research. The first component of this funding opportunity was to establish inter-professional collaborative teams of (1) investigators, health professional scientists, and clinicians who are (2) advised by patients, families, and communities and who are (3) supported by senior decision-makers to capitalize on one of the greatest assets in the healthcare sector, namely a highly educated and dedicated workforce with a clear mission to improve the health and well-being of the population. To foster innovative approaches to chronic disease prevention and management and to improve access to care for vulnerable populations, the goal was to mobilize grass-roots creativity. With 13 provincial and territorial healthcare systems, and a number of federally-run healthcare systems, the teams were to support cross-jurisdictional work to take advantage of natural experiments occurring in different provincial, territorial and federal systems and determine the conditions that influence success. Furthermore, the teams were required to show dedication to capacity building through training and mentoring opportunities and knowledge translation (KT).

We detail in this paper the methods, tools and activities of the FORGE AHEAD Program. Future publications will include details and results of the process evaluation and the outcome evaluation utilized within the FORGE AHEAD Program.

## Methods

### Aim

The TransFORmation of IndiGEnous PrimAry HEAlthcare Delivery (FORGE AHEAD): Community-driven Innovations and Strategic Scale-up Toolkits was funded by this initiative as a 5-year national research program that partners with Indigenous communities in Canada to improve chronic disease care and access to available resources by developing and evaluating community-driven, culturally-relevant primary healthcare models through a QI process [47]. The program was initiated in 2013 and consists of five key research objectives:

1. Assess the current healthcare delivery, funding models and best practices used in First Nations communities in Canada
2. Assess community and clinical readiness to address and adopt chronic disease care
3. Enhance patient access to available community resources for chronic disease care
4. Implement, evaluate and cost community and clinical QI initiatives to improve chronic disease management
5. Develop sustainment strategies and scale-up toolkits for improved chronic disease

### Research design and setting

**Fig. 1:**
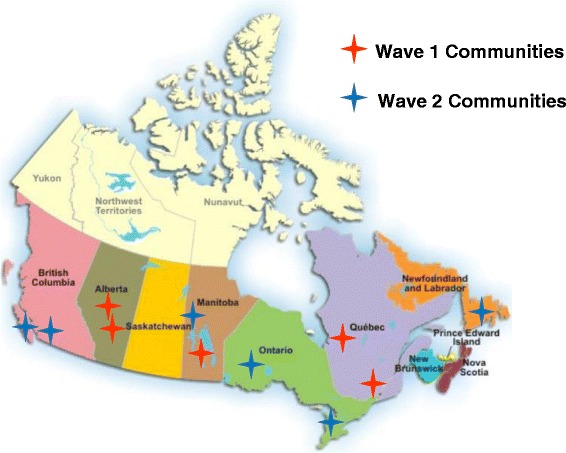
Map of partnering First Nations communities in the FORGE AHEAD Program separated by implementation wave

FORGE AHEAD partnered with 11 First Nations communities across six provinces (BC, AB, MB, ON, QC, NL) and three isolation levels (isolated, non-isolated, and remote-isolated/semi-isolated) (Fig. 1).

Communities were recruited into the program through self-expressed interest in response to regional and website sharing of program information and/or personal communication by investigators with pre-existing community partnerships. Community participation was confirmed by a signed research and financial agreements with each community. The program utilizes a pre-post mixed-methods observational design. Partnering communities serve as their own control in the pre-post design. The program is implemented in two waves in order to maximize community participation by adopting flexible timelines for recruitment and community engagement (Table 1). Participants consist of a Community Team and a Clinical Team that are each comprised of three to five consenting community members. All clinical team members are a part of the circle of care for patients with T2DM in their community.

**Table 1:** Community characteristics, including implementation wave, regional/provincial location and reported population

Region/Province	Wave	Community population^a^
Pacific – British Columbia	2	550
Pacific – British Columbia	2	1930
Prairie – Alberta	1	15,223
Prairie – Alberta	1	1934
Central – Manitoba	1	1767
Central – Manitoba	2	5548
Eastern – Ontario	2	550
Eastern – Ontario	2	756
Eastern – Quebec	1	1865
Eastern – Quebec	1	10,514
Atlantic – Newfoundland	2	865

^a^Population figures reported in the FORGE AHEAD Community Profile Survey and/or community website

### Ethics approval and guiding principles

Ethics approval for the FORGE AHEAD Program was received from the Western University Health Sciences Research Ethics Board ((#103895, approved June 17, 2013), the Health Research Ethics Board of Alberta (CHC-14-0054, approved December 1, 2014), the Cree Board of Health and Social Services of James Bay (#2014-DSP-03, approved October 2, 2014), Mi’kmaw Ethics Watch, Unama’ki College, Cape Breton University (approved January 29, 2014), and Mi’kmaq Confederacy Ethics Review Committee, Prince Edward Island (approved March 14, 2014). The FORGE AHEAD program is grounded in the guiding principles of: (1) community-based participatory research (CBPR) and the Ownership, Control, Access, and Possession® Principles [48] described by the First Nations Information Governance Centre; (2) capacity building; and (3) community-driven and culturally appropriate collaborative research, all of which guide the ethical conduct of the program. CBPR honours and reflects the communities’ involvement as full partners while ensuring the use of culturally appropriate processes and integrated KT [40, 49]. Communities partner with the research team to form collaborative partnerships whereby communities retain ownership of their data and have decision-making power throughout the program. The activities in FORGE AHEAD are linked to the ECCM that describes the inter-relationships of individual, community, population and health system factors in chronic disease prevention and care.

### Program governance

**Fig. 2:**
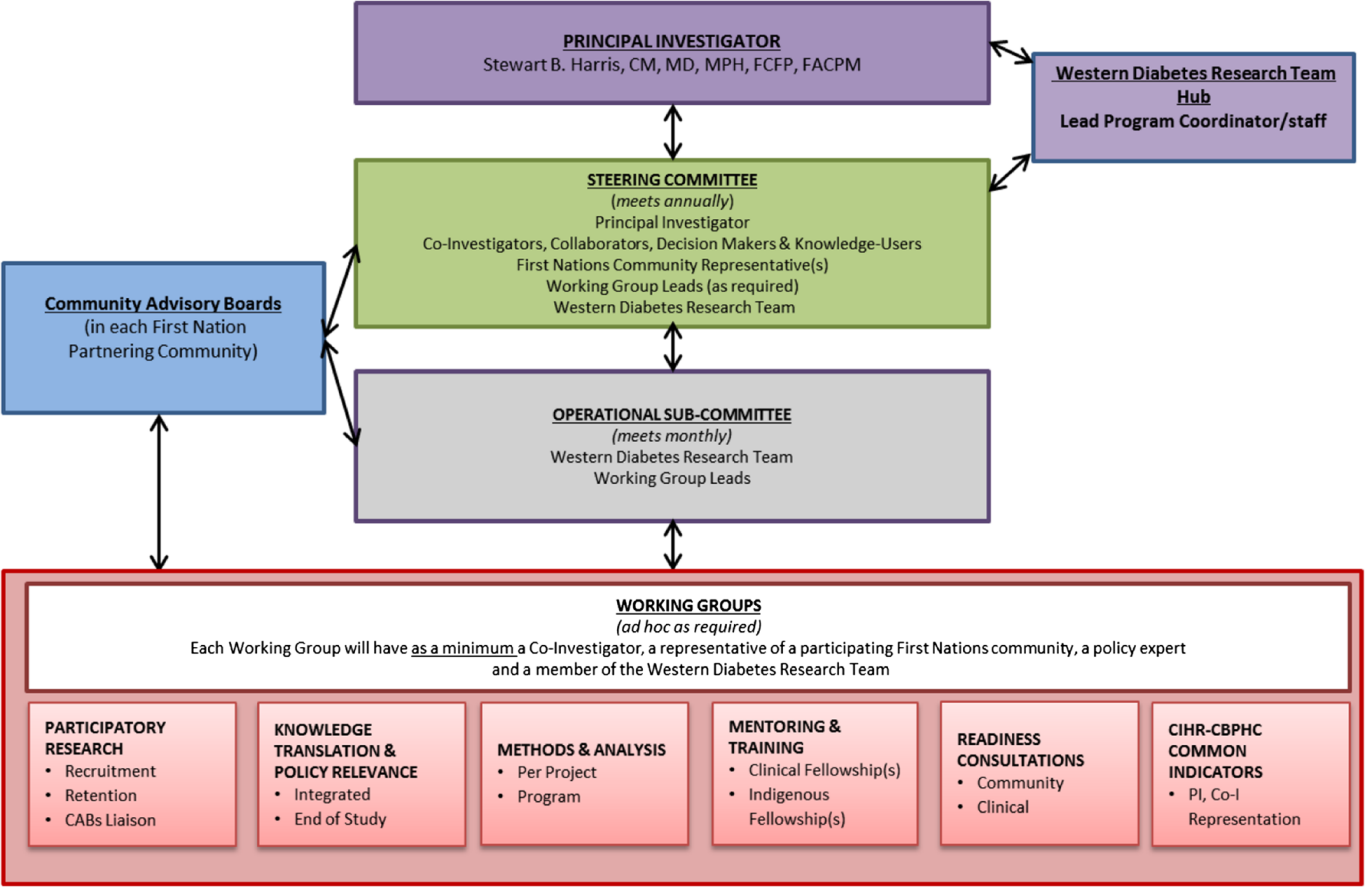
FORGE AHEAD governance structure schematic

Figure 2 displays a schematic of the governance structure of the program. Our strong multidisciplinary and cross-jurisdictional research team includes First Nations community representatives, Indigenous and non-Indigenous healthcare providers, clinician scientists, academic researchers, policy decision-makers, knowledge-users, and collaborators. This collaboration brings together nationally recognized leaders in the fields of clinical practice and/or research (T2DM in primary healthcare, chronic disease, nursing, nutrition, Indigenous research, participatory research, epidemiology, endocrinology, nephrology, KT, community action research, quality improvement methodology). All have experience in working collaboratively with First Nations communities.

### Program components

**Fig. 3:**
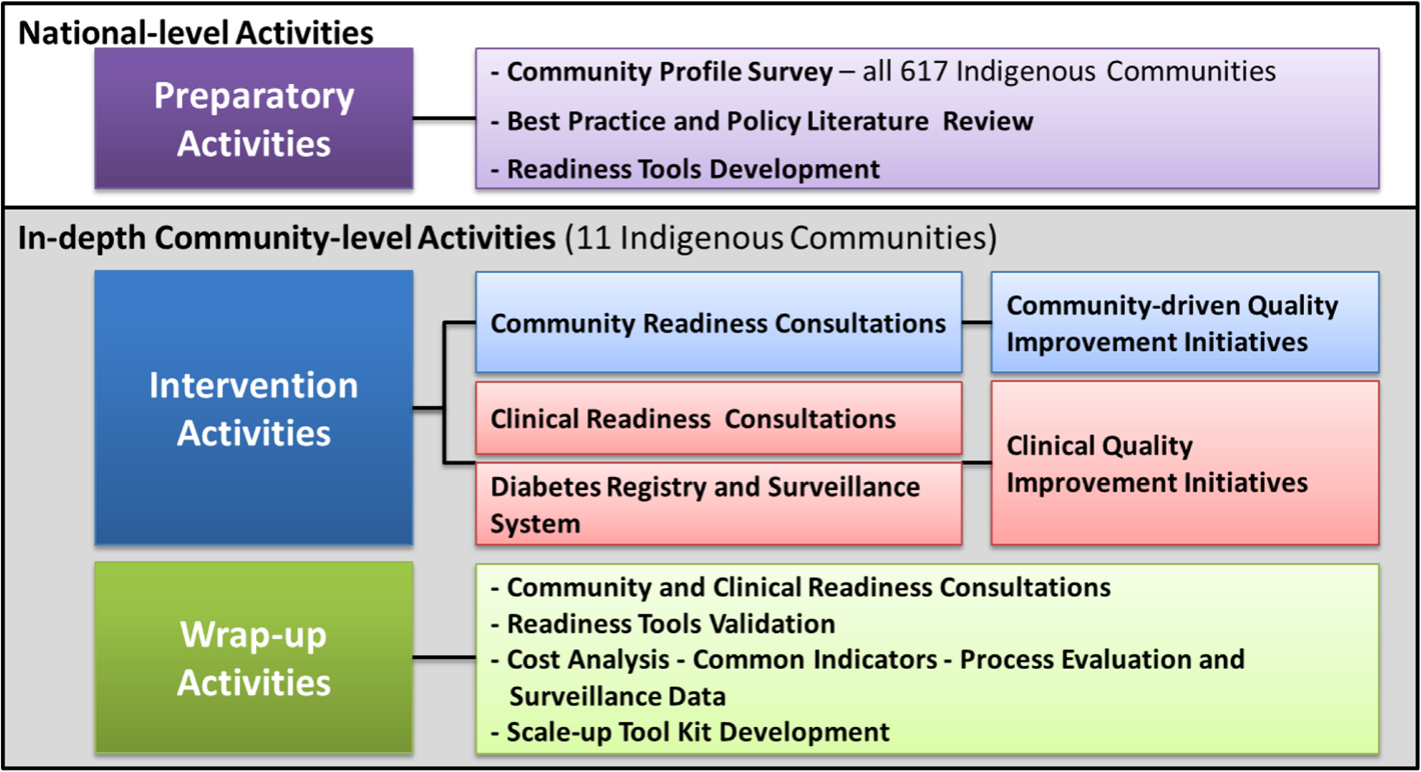
FORGE AHEAD Program activities by phases

The FORGE AHEAD Program is encompassed by a series of 10 inter-related and progressive activities separated into three components, namely, national-level preparatory activities, community-driven intervention activities and wrap-up/dissemination. Community participation spans a total of 18-months for all activities (Fig. 3).

#### National-level preparatory activities

All communities partnering in the program are asked to complete a Community Profile Survey (CPS) (Activity 1) to identify and describe current healthcare delivery, funding models, available infrastructure (nursing stations, healthcare centres, healing centres, hospitals, etc.), T2DM programs, and the number of health professionals working in their community, both part-time and full-time (Additional file 1). The CPS underwent regional tailored distribution to all 617 First Nations communities across Canada. The CPS activity was completed in June 2015 with the development and distribution of over 100 community and regional/national reports to all participating communities and key stakeholders including federal and regional government partners, and organizational partners. The CPS forms the basis for all other activities in the program for partnering communities.

The Best Practice and Policy Literature Review (Activity 2) was also completed in 2015 and involved a synthesis of healthcare policy up to 2008 relevant to Indigenous communities to identify and describe existing healthcare policies affecting Indigenous communities and best practices developed by and for Indigenous communities. A systematic review of peer-reviewed and grey literature was conducted and published [50]. The knowledge gained from the literature review served as a platform for all subsequent program activities.

**Fig. 4:**
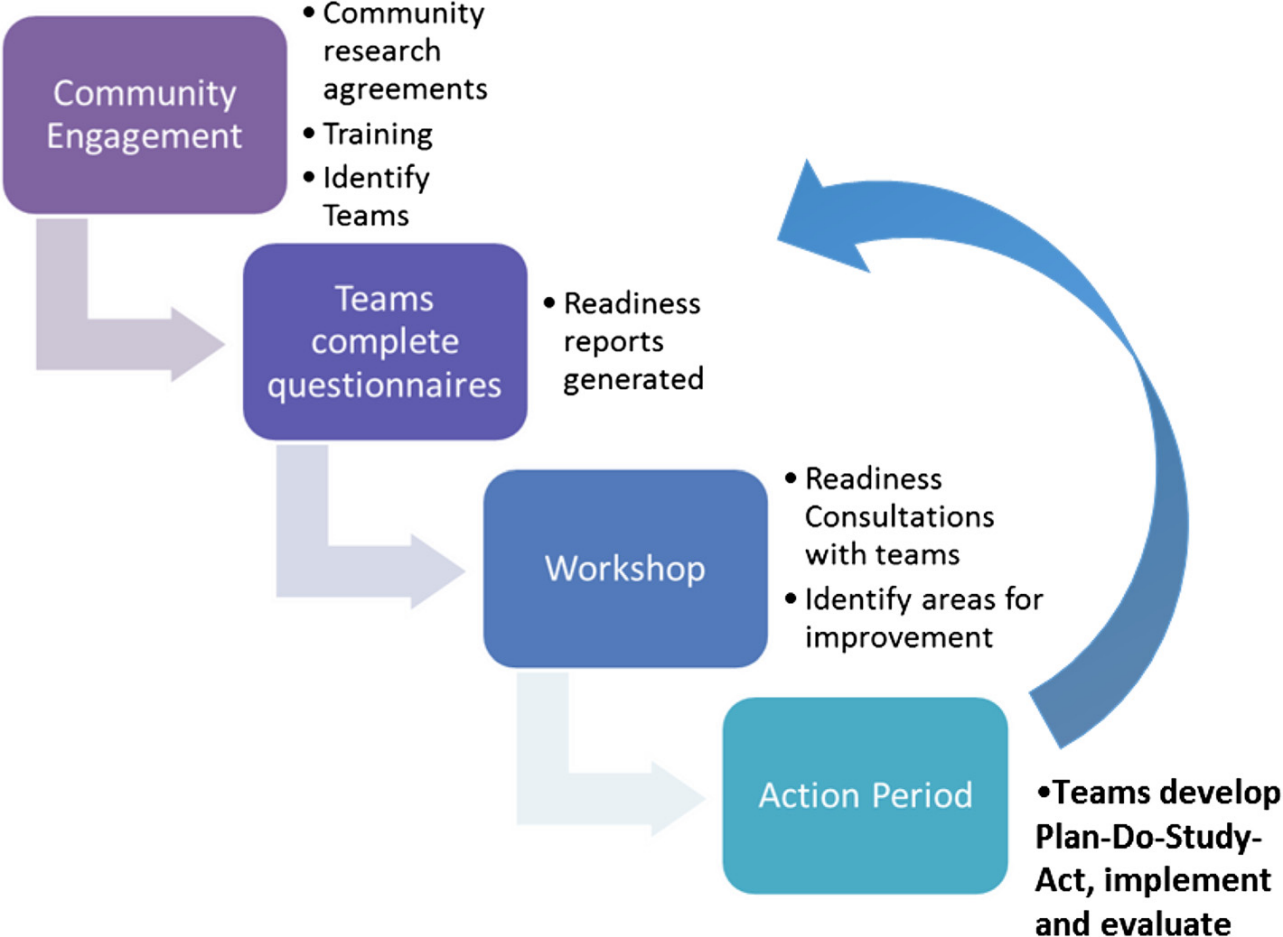
Readiness consultation and QI initiatives process

The Community Readiness Model (CRM) for community change integrates a community’s culture, resources, level of knowledge, and support to determine the level of readiness to address an issue [51]. Readiness is the degree to which a community is prepared to take action on an issue. Readiness is issue-specific, measureable, has multiple dimensions and can improve [51]. Proponents of the CRM argue that matching an intervention to a community’s level of readiness is absolutely essential for success and sustainment of an intervention [51, 52]. The CRM empowers the community to take ownership of their particular concern, fortifying their capacity to move forward [53]. As part of the FORGE AHEAD Program, the team sought to develop and validate both a Community Readiness Consultation Tool and a Clinical Readiness Consultation Tool (Naqshbandi Hayward M, Mequanint S, Paquette-Warren J, Bailie R, Chirila A, Dyck R, Green M, Hanley A, Tompkins JW, Harris SB. The FORGE AHEAD Clinical Readiness Consultation Tool: a validated tool to assess clinical readiness for chronic disease care mobilization in Canada’s First Nations. Submitted to BMC Health Serv Res. 2016) (Activities 3 and 4) (Fig. 4). The two tools were aligned for use by the two teams participating in the program from each community; the Community Team and the Clinical Team. The aim of the tools is to rank the level of community and clinical team readiness to develop, adopt and evaluate chronic disease care initiatives. After a comprehensive literature search, the CRM tool [54] and the Audit and Best Practice for Chronic Disease Systems Assessment Tool [55, 56] were adapted by a Working Group consisting of academic researchers, clinicians, and First Nations community representatives to tailor the tools for use in the Canadian First Nations primary healthcare setting and as stand-alone tools that do not require resources external to the communities for their completion. Both tools were piloted with a group of co-investigators and Indigenous and non-Indigenous community members from the 11 partnering communities involved in the planning and development of the FORGE AHEAD Research Program proposal. All feedback, comments and recommendations were integrated into the final version of the tools that are being used during the intervention phase.

#### Community-driven intervention activities

Community Readiness Consultation (Activity 5), the first step of the intervention, uses the Community Readiness Consultation Tool developed during the preparatory phase to facilitate discussions within the Community Team of three to five members (community primary prevention programs, community clinical care programs, traditional and health leadership, or community-at-large living with diabetes) to begin brainstorming ideas for the development of chronic disease initiatives. Similarly, the Clinical Readiness Consultation (Activity 6) utilizes the Clinical Readiness Consultation Tool within the Clinical Team. After each team member individually completes their respective tool, the Western research team develops aggregated reports that summarize the results across the team members. Community Facilitators, hired and trained by the Western research team, guide their teams in completing the tools and lead discussions at round-table meetings with each of their teams to review, share and discuss summarized results. Each team has an opportunity to change the scores on their reports based on team consensus before proceeding to use the report to identify key factors and priorities for the development of chronic disease innovations. The readiness tools are completed by each team member three times during the course of the program. Each time, aggregated reports are developed and reviewed by each team. The ultimate aim of these reports is to facilitate discussions by each team to align their chronic disease initiatives to their degree of readiness, thereby increasing the likelihood that the initiatives will lead to improvements.

Community-driven (Activity 7) and Clinical (Activity 8) QI Initiatives includes a series of three workshops (1–2 days), separated by 3-month action periods.

Each workshop involves three parts: (1) plenaries with expert presenters for all teams to learn about priority topics as a group; (2) individual team sharing of key community strengths, progress and challenges to enable cross community learning and support; and (3) breakout sessions where individual teams are allotted dedicated time together to discuss their readiness reports and plan their QI initiatives using the Model for Improvement [57, 58] including Plan-Do-Study-Act (PDSA) cycles. Due to funding limitations, only the first workshop for each team in each wave of implementation is held face-to-face in London, Ontario. The remaining two workshops are held by videoconferencing software with each community partner joining from their own communities. During the action periods, support is provided by the Western research team to respond to communities’ needs for ongoing training, facilitation support, documentation and resources to assist teams through their PDSA cycles. Community Facilitators play the role of the community champion guiding their teams through the PDSA cycles during each of the action periods.

Building on previously successful QI initiatives, the program includes the development of a registry [24, 59]. Identification of patients diagnosed with T2DM is a critical first step to target interventions for these patients and carry out follow-up surveillance of T2DM measures. The Diabetes Registry and Surveillance System (Activity 9) focuses on the development of a diabetes registry enumerating all adult (≥18 years) diagnoses of T2DM in each partnering community. Registries securely house name, gender, year of birth, diagnosis date, health card number, and Band status number. Community members have the option of opting out of the registry. Activity 9 also includes a web-based surveillance system [47] that securely houses clinical information for all patients listed on the community’s diabetes registry who have not opted out of the system. The web-based system includes built-in tools and clinical reminders to support improvement of diabetes care. Community Data Coordinators, hired from each partnering community and trained by the Western research team, populate the system. Access to the registry and surveillance system is determined by individual Community Advisory Boards. The research team only has access to the aggregated, de-identified data from all participating communities. To avoid duplication of resources and workload, communities that have pre-existing surveillance systems or electronic medical records and choose to utilize their own systems as part of the program are supported in adapting their systems to ensure the collection of all outcomes related to T2DM.

#### Wrap-up and dissemination activities

Post Community Readiness and post Clinical Readiness Consultations will be conducted to assess community readiness and clinical readiness at program’s end. In addition, both readiness tools will undergo a validation process to develop and implement innovations in improving chronic care prevention and management in First Nations communities.

To evaluate the success of the FORGE AHEAD Program, a comprehensive (process and outcome) evaluation will be used and facilitated by the development of a logic model of the program. The logic model development will be guided by the worksheets of the Diabetes Evaluation Framework for Innovative National Evaluations (DEFINE) [46, 60] and will serve as a tool to describe key contextual factors that influence the effectiveness of the program, identify evidence needed to build knowledge about successful interventions activities and implementation strategies, and help capture what and how key elements work across multiple settings. Constructs included in the DEFINE health determinant schematic and associated priority indicator set (patient, healthcare delivery, organization of care, and environment levels) will be considered to help identify important aspects to measure, including the interaction among the program activities, implementation processes, adaptability, setting, and the people involved. Capturing evidence about the adaptation, implementation and impact of program activities, assessing intended versus unintended activities, determining which activities were perceived as most effective and why within specific and varied settings will be essential to developing the scale-up toolkits.

Analyses (Activity 10) will include a cost analysis to determine the cost of implementing the intervention (i.e. costs with organizing and operating the initiative, and use of resources) in First Nations communities. It is important to be able to quantify the cost of the quality improvement initiatives to inform sustainability and scale-up initiatives. In-depth process evaluations, including key stakeholder interviews (e.g. Community Facilitators, Community Team members, Clinical Team members, etc.) will be held to gain knowledge on their perceptions of various QI activities/application/benefits. Process evaluation will also include a detailed review of all program documentation to identify details about program activities and the implementation process, and to learn about the experience of those who participated in the program and their perceptions of the impact of the research program in their individual communities. The Western research team will work with partnering communities to determine the completeness of their surveillance system. Support will be provided to the Community Data Coordinators to update the system as needed to support the evaluation of the intervention.

FORGE AHEAD will produce a toolkit of tested tools and strategies that can be successfully implemented, sustained and used for diabetes and other chronic diseases in First Nations communities across Canada. Scale-up toolkits will be developed by integrating tools and best practices between Health Canada and the FORGE AHEAD Research Program. Toolkits and all results of the program will be disseminated to each partnering community prior to undertaking national and international dissemination efforts.

### KT and engagement

The principles of CBPR support integrated and end-of-program KT with communities involved as equal partners in the research process [61]. Community partners, policymakers, and academic and organizational partners were involved in setting the objectives of this program, collecting the data, interpreting the results, and determining dissemination and implementation strategies within their own stakeholder groups and to a broader audience [61]. Integrating key stakeholders fosters a sense of ownership over the knowledge creation process, increasing the probability that research findings will be acted upon in the relevant settings [62]. Project implementation and results will be continually shared with all key stakeholders through community gatherings, presentations to Chief and Councils and at Steering Committee meetings. KT plans will be developed in collaboration with each community through their community advisory boards and across communities to evaluate barriers and optimize facilitators, knowledge sharing and discussion. Early investment in integrated KT to reach diverse stakeholders and audiences in community-based research to support diabetes QI in First Nations communities has enabled the integration of traditional and evidence-based knowledge in progressive projects. A mix of communication and social media, including a FORGE AHEAD website (www.tndms.ca/forgeahead/) that houses resources, program documents and support documents, a FORGE AHEAD Facebook page (www.facebook.com/FAProgram), and quarterly newsletters have been a critical platform for updating all team members and building camaraderie.

## Study status

The FORGE AHEAD Program is in its fourth year and still being implemented. A chronic disease approach is promoted and is being laid through the development of QI innovations by community and clinical teams in partnering communities. Successful models of care are being rapidly identified and tested using the PDSA cycle, and the conditions necessary for sustainment and scale-up of successful innovations are being investigated throughout the process. Planning for and implementing integrated and program-end KT is ahead of schedule as well as the development the FORGE AHEAD Scale-up Toolkits.

Comprehensive data on the recruitment and retention of partnering communities, and community and clinical team members is not yet available. At program end, complete evaluation data will be available detailing the use of all FORGE AHEAD tools, including the Community and Clinical Readiness tools, the First Nations Diabetes Sentinel Surveillance System, and all QI tools used to facilitate the development of community-driven initiatives. The impact of these tools on health-related outcomes, the cost of implementing the various activities within the program, and detailed process evaluations including the logic model, participant observation, project documentation reviews and community interviews will inform further revisions to all tools as well as inform best practices for the successful implementation and scale-up of the program.

## Discussion

Indigenous communities in Canada represent a unique challenge for our healthcare system with soaring rates of chronic diseases, a wide array of funding and service models, and distinctive barriers to providing optimal care. Despite recent efforts to fund more chronic disease-oriented initiatives and programs, rates of chronic diseases such T2DM continue to rise and the gap between Indigenous and non-Indigenous peoples continues to widen. In Indigenous settings, community engagement and participation is crucial to identifying and developing successful and sustainable innovations that can improve access to essential and culturally relevant chronic disease-focused primary healthcare services with the ultimate goal of reducing inequities in health.

The FORGE AHEAD Program set out to promote and evaluate the development of community-driven, culturally relevant, primary healthcare models that enhance chronic disease management and appropriate access to available services in First Nations communities across Canada. The program is implemented in two waves with 11 First Nations communities across seven provinces and utilizes a participatory research approach recognizing communities as true partners. As partners, their contribution to the program from the inception of the research design to implementation of the program activities is acknowledged and appreciated as a critical grass-roots perspective that will help bring to the centre of efforts the Indigenous voice that is necessary in order to succeed.

Some of the practical challenges to this type of work are the cost of developing and implementing such programs in remote locations, the lack of resources available within the community, and the long timelines needed to establish rapport and trusting relationships. The FORGE AHEAD team has been fortunate in securing additional funding to support the program and with the expertise housed on our experienced and skilled team, the program is being successfully implemented. It is important to note that in-kind contributions by many of the FORGE AHEAD Program team members from co-investigators, collaborators, knowledge-users and decision-makers, and elders, leaders and key contacts in partnering communities have helped to build a meaningful and solid foundation as FORGE AHEAD progresses into its final year of implementation. Limited funding has restricted the number and type of in-person collaborations; however, new electronic platforms have been successfully employed to build and sustain strong relationships with community partners and enhanced the capacity of this team, which is spread across the country, to function virtually. Our KT efforts include a Facebook site designed to build camaraderie and support, a website that houses all program materials and documents accessible by all team members at any time, program newsletters that include spotlights of key successes and updates, and the use of teleconferencing and videoconferencing technology.

Critical factors related to the on-going success of the program to date have been identified, including the large team’s leadership and depth of interdisciplinary research/practice, key community personnel who are champions in their communities leading the QI work, the capacity to develop new partnerships and obtain additional funding to ensure true participatory research, enhancement of capacity building and training opportunities, and networking and incorporating a wide variety of integrated and program-end KT activities. Preliminary process evaluation results reveal the important role of the Community Facilitators and Community Data Coordinators who are committed, skilled and respected leaders in their communities. The strength of the FORGE AHEAD Program lays within the team – the commitment, the leadership, the experience and the diverse skill set of people who are passionate about improving the lives of people living with diabetes by decreasing the health, clinical and financial burden of diabetes in Indigenous communities.

The outcomes of the FORGE AHEAD Program, its related cost and the subsequent policy recommendations have the potential to support the redesign of chronic disease care and management in First Nations communities across Canada. Results may significantly affect future chronic disease policy decisions pertaining to First Nations communities.

## Abbreviations

CBPR, community-based participatory research; CCM, chronic care model; CPS, community profile survey; CRM, community readiness model; DEFINE, diabetes evaluation framework for innovative national evaluations; ECCM, expanded chronic care model; FORGE AHEAD, transformation of Indigenous primary healthcare delivery: community-driven innovations and strategic scale-up toolkits; HbA1C, glycosylated haemoglobin; KT, knowledge translation; PDSA, plan-do-study-act; QI, quality improvement; T2DM, type 2 diabetes mellitus.

## Additional file

Additional file 1: FORGE AHEAD: Community Profile Survey. (DOCX 144 kb)
