# Supplementary material for: Developing community-driven quality improvement initiatives to enhance chronic disease care in Indigenous communities in Canada: the FORGE AHEAD program protocol
Source: Health Res Policy Syst. 2016 Jul 26;14:55. doi: 10.1186/s12961-016-0127-y (PMC4960663; doi:10.1186/s12961-016-0127-y)
Supplement: Additional file 1: — FORGE AHEAD: Community Profile Survey. (DOCX 144 kb) [file 12961_2016_127_MOESM1_ESM.docx]

**FORGE AHEAD: Community Profile Survey**

The Transformation of Indigenous Primary Healthcare Delivery (FORGE AHEAD) Research Program team invites you to participate in this national Community Profile Survey to learn more about the healthcare delivery, funding models, and diabetes programs currently available in First Nations communities. All First Nations communities across Canada are invited to participate in this survey, which is the first of many projects that are part of the five year FORGE AHEAD Research Program. FORGE AHEAD is designed with and for First Nations communities to improve care for people with diabetes. For more information about FORGE AHEAD, please visit our website at <http://www.tndms.ca/forgeahead.html>.

The person who is most familiar with how healthcare is organized and operates in your community should fill in the survey. Typically, this can include the Health Director or Nurse-in-charge or band council leader. **Only one survey per community should be completed.** There are no right or wrong answers.

.

**SECTION 1: YOUR COMMUNITY PROFILE**

**Please complete your community’s name and other information below.**

|  | |  | | | |  | | | | | | | | | | | | | | | |  |  |  |  |  |  |  |  |
| --- | --- | --- | --- | --- | --- | --- | --- | --- | --- | --- | --- | --- | --- | --- | --- | --- | --- | --- | --- | --- | --- | --- | --- | --- | --- | --- | --- | --- | --- |
| **1.** | | **Community Name:** | | | |  | | | | | | | | | | | | | | | |  |  |  |  |  |  |  |  |
| 2. | | Address: | | | |  | | | | | | | | | | | | | | | |  |  |  |  |  |  |  |  |
| 3. | | **Total** estimated community population: | | | | | | | | | | a) Total | | | | |  | | | | |  |  |  |  |  |  |  |  |
|  | |  | | | | | | | | | | b) Male | | | | |  | | | | |  |  |  |  |  |  |  |  |
|  | |  | | | | | | | | | | c) Female | | | | |  | | | | |  |  |  |  |  |  |  |  |
| 4. | | Estimated **on-reserve** population: | | | | | | | | | | a) Total | | | | |  | | | | |  |  |  |  |  |  |  |  |
|  | |  | | | | | | | | | | b) Male | | | | |  | | | | |  |  |  |  |  |  |  |  |
|  | |  | | | | | | | | | | c) Female | | | | |  | | | | |  |  |  |  |  |  |  |  |
| 5. | | Estimated **off-reserve** population: | | | | | | | | | | a) Total | | | | |  | | | | |  |  |  |  |  |  |  |  |
|  | |  | | | | | | | | | | b) Male | | | | |  | | | | |  |  |  |  |  |  |  |  |
|  | |  | | | | | | | | | | c) Female | | | | |  | | | | |  |  |  |  |  |  |  |  |
| 6. | What is your community’s isolation level? | | | | | | | | | | | | | | | | | | | | | | | | |  |  | |  |
|  | 🞎 | | Non-isolated *(road access less than 90 km from nearest physician services)* | | | | 🞎 | | Semi-isolated *(road access greater than 90 km from nearest physician services)* | | | | | | 🞎 | Isolated *(flights, good telephone service, no road access)* | | | | | 🞎 | | Remote isolated  *(no scheduled flights, no road access, and minimal telephone and radio)* | | | 🞎 | Don’t Know | |  |
| 7. | | What type of health service centre(s) is available in your community? PLEASE MARK ALL THAT APPLY. | | | | | | | | | | | | | | | | | | | | | | | | | | |  |
|  | | 🞎 | | Nursing Station | 🞎 | | | Health Office | | | | | 🞎 | Health Centre | | | | | | 🞎 | | Community Hospital | | 🞎 | None: Go to Question #10 | | | | |
| 8. | | Are there any other communities (e.g. satellite communities) that are served by your community’s health service centre(s)? | | | | | | | | | | | | | | | | | | | | | | | | | |  |  |
|  | | 🞎 | | Yes | | | | | | 🞎 | No | | | | | | |  | | | | | | | | | |  |  |
|  | | If ‘YES’, please provide the names of each community served. | | | | | | | | | | | | | | | | | | | | | | | | | |  |  |
|  | |  | |  | | | | | | | | | | | | | |  |  | | | | | | | | |  |  |
|  | |  | |  | | | | | | | | | | | | | |  |  | | | | | | | | |  |  |
|  | |  | |  | | | | | | | | | | | | | |  |  | | | | | | | | |  |  |

| 9. | Does the health service centre(s) available in your community have any of the following? PLEASE MARK ALL THAT APPLY. | | | | | | | | | | | | | | | | |  |  |  |  |
| --- | --- | --- | --- | --- | --- | --- | --- | --- | --- | --- | --- | --- | --- | --- | --- | --- | --- | --- | --- | --- | --- |
|  | 🞎 | | | Computer | | 🞎 | | Internet access | | | | | 🞎 | | an electronic medical record (EMR) used for charting | | |  |  |  |  |
| 10. | Does your community have a searchable list of individuals with Type 2 diabetes (diabetes registry)? | | | | | | | | | | | | | | | | |  |  |  |  |
|  | 🞎 | | | Yes | | 🞎 | | No: Go to Question #14 | | | | |  | | | | |  |  |  |  |
| 11. | When was the last time your community’s diabetes registry was updated? | | | | | | | | | | | | | | | | |  |  |  |  |
|  |  | Month | | |  | | Year | | | | |  | |  | | | | | | |  |
| 12. | What is the format of the diabetes registry in your community? | | | | | | | | | | | | | | | | | | | | |
|  | 🞎 | | Electronic-based | | | | | | 🞎 | | Paper-based | | | | | | | |  |  |  |
| 13. | Where is the community’s diabetes registry housed? | | | | | | | | | | | | | | | | | | | | |
|  | 🞎 | | Within the Community | | | | | | | 🞎 | Neighbouring Aboriginal Community | | | | | 🞎 | Non-Aboriginal Urban Centre | | |  |  |
| 14. | Does your community have an electronic/computer system for tracking and monitoring patient’s diabetes clinical information (diabetes surveillance system)? | | | | | | | | | | | | | | | | | | | | |
|  | 🞎 | | | Yes | | 🞎 | | No | | | | | | | | | | | | | |
| 15. | What is the total estimated number of adults with type 2 diabetes in your community? ______________________ | | | | | | | | | | | | | | | | | | | | |
| 16. | What is your current job title? ________________________________________ | | | | | | | | | | | | | | | | | | | | |
|  |  | | | | | | | | | | | | | | | | | | | | |

**SECTION 2: HEALTHCARE PROFESSIONALS**

1. Please check the appropriate box to indicate the **availability** (on-site, indirect, visiting, off-site) of **healthcare professionals** in your community. Where applicable, please provide the estimated **frequency of visits** for visiting healthcare professionals and **distance** for off-site healthcare professionals.

| **HEALTHCARE PROFESSIONALS** | **NOT APPLICABLE** (i.e., not available on- or off-site) | **USUALLY AVAILABLE**  **ON-SITE** (i.e., live and work in the community) | **INDIRECTLY AVAILABLE** (i.e., through phone/internet, Telehealth) | **ONLY VISITING**  (e.g., fly-in, mobile truck) | | **ONLY AVAILABLE OFF-SITE**  (i.e., community members required to travel) | | | | | |
| --- | --- | --- | --- | --- | --- | --- | --- | --- | --- | --- | --- |
|  |  |  |  | **Estimated # of visits per**  **(month OR year):** | | **Neighbouring Aboriginal Community** | **Non-Aboriginal Town or Urban Centre** | **Estimated Distance:** | | | |
|  |  |  |  | month | year |  |  | <20km | 20-40km | 40-80km | >80km |
| Health Director | 🞎 | 🞎 | 🞎 |  |  | 🞎 | 🞎 | 🞎 | 🞎 | 🞎 | 🞎 |
| Nurse-in-charge | 🞎 | 🞎 | 🞎 |  |  | 🞎 | 🞎 | 🞎 | 🞎 | 🞎 | 🞎 |
| Family physician | 🞎 | 🞎 | 🞎 |  |  | 🞎 | 🞎 | 🞎 | 🞎 | 🞎 | 🞎 |
| Nurse Practitioner | 🞎 | 🞎 | 🞎 |  |  | 🞎 | 🞎 | 🞎 | 🞎 | 🞎 | 🞎 |
| Public Health Nurse | 🞎 | 🞎 | 🞎 |  |  | 🞎 | 🞎 | 🞎 | 🞎 | 🞎 | 🞎 |
| Community Health Nurse | 🞎 | 🞎 | 🞎 |  |  | 🞎 | 🞎 | 🞎 | 🞎 | 🞎 | 🞎 |
| Home Care Nurse | 🞎 | 🞎 | 🞎 |  |  | 🞎 | 🞎 | 🞎 | 🞎 | 🞎 | 🞎 |
| Community Health Representative | 🞎 | 🞎 | 🞎 |  |  | 🞎 | 🞎 | 🞎 | 🞎 | 🞎 | 🞎 |
| Personal Care Workers | 🞎 | 🞎 | 🞎 |  |  | 🞎 | 🞎 | 🞎 | 🞎 | 🞎 | 🞎 |
| Diabetes Nurse Educator | 🞎 | 🞎 | 🞎 |  |  | 🞎 | 🞎 | 🞎 | 🞎 | 🞎 | 🞎 |
| Community Diabetes Educator | 🞎 | 🞎 | 🞎 |  |  | 🞎 | 🞎 | 🞎 | 🞎 | 🞎 | 🞎 |
| Dietitian | 🞎 | 🞎 | 🞎 |  |  | 🞎 | 🞎 | 🞎 | 🞎 | 🞎 | 🞎 |
| Social Worker | 🞎 | 🞎 | 🞎 |  |  | 🞎 | 🞎 | 🞎 | 🞎 | 🞎 | 🞎 |
| Mental Health Therapist | 🞎 | 🞎 | 🞎 |  |  | 🞎 | 🞎 | 🞎 | 🞎 | 🞎 | 🞎 |
| Pharmacist | 🞎 | 🞎 | 🞎 |  |  | 🞎 | 🞎 | 🞎 | 🞎 | 🞎 | 🞎 |
| Dentist | 🞎 | 🞎 | 🞎 |  |  | 🞎 | 🞎 | 🞎 | 🞎 | 🞎 | 🞎 |
| Dental Hygienist | 🞎 | 🞎 | 🞎 |  |  | 🞎 | 🞎 | 🞎 | 🞎 | 🞎 | 🞎 |
| Traditional Healers / Elders | 🞎 | 🞎 | 🞎 |  |  | 🞎 | 🞎 | 🞎 | 🞎 | 🞎 | 🞎 |
| Cultural Coordinators | 🞎 | 🞎 | 🞎 |  |  | 🞎 | 🞎 | 🞎 | 🞎 | 🞎 | 🞎 |

1. Please check the appropriate box to indicate the **availability** (on-site, indirect, visiting, off-site) of **medical specialists** in your community. Where applicable, please provide the estimated **frequency of visits** for visiting specialists and **distance** for off-site specialists.

| **MEDICAL SPECIALISTS** | **USUALLY AVAILABLE**  **ON-SITE** (i.e., live and work in the community) | **INDIRECTLY AVAILABLE** (i.e., through phone/internet, Telehealth) | **ONLY VISITING**  (e.g., fly-in, mobile truck) | | **ONLY AVAILABLE OFF-SITE**  (i.e., community members required to travel) | | | | | |
| --- | --- | --- | --- | --- | --- | --- | --- | --- | --- | --- |
|  |  |  | **Estimated # of visits per**  **(month OR year):** | | **Neighbouring Aboriginal Community** | **Non-Aboriginal Town or Urban Centre** | **Estimated Distance:** | | | |
|  |  |  | month | year |  |  | <20km | 20-40km | 40-80km | >80km |
| Endocrinologist | 🞎 | 🞎 |  |  | 🞎 | 🞎 | 🞎 | 🞎 | 🞎 | 🞎 |
| Wound Care Specialist | 🞎 | 🞎 |  |  | 🞎 | 🞎 | 🞎 | 🞎 | 🞎 | 🞎 |
| Podiatrist /Chiropodist | 🞎 | 🞎 |  |  | 🞎 | 🞎 | 🞎 | 🞎 | 🞎 | 🞎 |
| Physiotherapist | 🞎 | 🞎 |  |  | 🞎 | 🞎 | 🞎 | 🞎 | 🞎 | 🞎 |
| Optometrist | 🞎 | 🞎 |  |  | 🞎 | 🞎 | 🞎 | 🞎 | 🞎 | 🞎 |
| Ophthalmologist | 🞎 | 🞎 |  |  | 🞎 | 🞎 | 🞎 | 🞎 | 🞎 | 🞎 |
| Cardiologist/Internist | 🞎 | 🞎 |  |  | 🞎 | 🞎 | 🞎 | 🞎 | 🞎 | 🞎 |
| Nephrologist | 🞎 | 🞎 |  |  | 🞎 | 🞎 | 🞎 | 🞎 | 🞎 | 🞎 |
| Neurologist | 🞎 | 🞎 |  |  | 🞎 | 🞎 | 🞎 | 🞎 | 🞎 | 🞎 |
| Vascular Surgeon | 🞎 | 🞎 |  |  | 🞎 | 🞎 | 🞎 | 🞎 | 🞎 | 🞎 |
| Orthopedic | 🞎 | 🞎 |  |  | 🞎 | 🞎 | 🞎 | 🞎 | 🞎 | 🞎 |
| Plastic Surgeon | 🞎 | 🞎 |  |  | 🞎 | 🞎 | 🞎 | 🞎 | 🞎 | 🞎 |
| Psychiatrist | 🞎 | 🞎 |  |  | 🞎 | 🞎 | 🞎 | 🞎 | 🞎 | 🞎 |
| Pediatrician | 🞎 | 🞎 |  |  | 🞎 | 🞎 | 🞎 | 🞎 | 🞎 | 🞎 |
| Other (specify):  ______________________________ | 🞎 | 🞎 |  |  | 🞎 | 🞎 | 🞎 | 🞎 | 🞎 | 🞎 |
| Other (specify):  ______________________________ | 🞎 | 🞎 |  |  | 🞎 | 🞎 | 🞎 | 🞎 | 🞎 | 🞎 |
| Other (specify):  ______________________________ | 🞎 | 🞎 |  |  | 🞎 | 🞎 | 🞎 | 🞎 | 🞎 | 🞎 |

**IF THERE IS NO HEALTH CARE CENTER AVAILABLE IN YOUR COMMUNITY, PLEASE GO TO SECTION 3 ON PAGE 8.**

1. Please indicate the number of available and filled FTE positions, and employment/funding details of all healthcare providers that are **available on-site or visiting** for members of your community. Please DO NOT indicate peoples’ names – only their positions. NOTE - *A FTE (Full-time equivalent) of 1.0 means that the person is a full-time worker; while a FTE of 0.5 means that the worker is half-time). Examples are provided below.*

| **Position** | Number of available FTE positions | Number of filled FTE positions | **Funding Source** | | | | | **Pay Structure** | | | | |
| --- | --- | --- | --- | --- | --- | --- | --- | --- | --- | --- | --- | --- |
|  |  |  | Provincial | Federal | Community | Tribal Council | Other (specify):  _______________ | Salary | Contract/Per diem | Fee-for-Service | Honorarium | Other (specify):  _______________ |
| ***Example: Nurse-In-Charge*** | *1.5* | *1* | *🗷* | *🞎* | *🗷* | *🞎* | *🞎* | *🗷* | *🞎* | *🞎* | *🞎* | *🞎* |
| ***Example: Dietitian*** | *1* | *0.2* | *🞎* | *🞎* | *🗷* | *🞎* | *🞎* | *🞎* | *🗷* | *🞎* | *🞎* | *🞎* |
| Family physician |  |  | 🞎 | 🞎 | 🞎 | 🞎 | 🞎 | 🞎 | 🞎 | 🞎 | 🞎 | 🞎 |
| Nurse-in-charge |  |  | 🞎 | 🞎 | 🞎 | 🞎 | 🞎 | 🞎 | 🞎 | 🞎 | 🞎 | 🞎 |
| Health Director |  |  | 🞎 | 🞎 | 🞎 | 🞎 | 🞎 | 🞎 | 🞎 | 🞎 | 🞎 | 🞎 |
| Nurse Practitioner |  |  | 🞎 | 🞎 | 🞎 | 🞎 | 🞎 | 🞎 | 🞎 | 🞎 | 🞎 | 🞎 |
| Public Health Nurse |  |  | 🞎 | 🞎 | 🞎 | 🞎 | 🞎 | 🞎 | 🞎 | 🞎 | 🞎 | 🞎 |
| Community Health Nurse |  |  | 🞎 | 🞎 | 🞎 | 🞎 | 🞎 | 🞎 | 🞎 | 🞎 | 🞎 | 🞎 |
| Home Care Nurse |  |  | 🞎 | 🞎 | 🞎 | 🞎 | 🞎 | 🞎 | 🞎 | 🞎 | 🞎 | 🞎 |
| Community Health Representative |  |  | 🞎 | 🞎 | 🞎 | 🞎 | 🞎 | 🞎 | 🞎 | 🞎 | 🞎 | 🞎 |
| Personal Care Workers |  |  | 🞎 | 🞎 | 🞎 | 🞎 | 🞎 | 🞎 | 🞎 | 🞎 | 🞎 | 🞎 |
| Community Diabetes Educator |  |  | 🞎 | 🞎 | 🞎 | 🞎 | 🞎 | 🞎 | 🞎 | 🞎 | 🞎 | 🞎 |
| Diabetes Nurse Educator |  |  | 🞎 | 🞎 | 🞎 | 🞎 | 🞎 | 🞎 | 🞎 | 🞎 | 🞎 | 🞎 |
| Dietitian |  |  | 🞎 | 🞎 | 🞎 | 🞎 | 🞎 | 🞎 | 🞎 | 🞎 | 🞎 | 🞎 |
| Social Worker |  |  | 🞎 | 🞎 | 🞎 | 🞎 | 🞎 | 🞎 | 🞎 | 🞎 | 🞎 | 🞎 |
| Mental Health Therapist |  |  | 🞎 | 🞎 | 🞎 | 🞎 | 🞎 | 🞎 | 🞎 | 🞎 | 🞎 | 🞎 |
| Pharmacist |  |  | 🞎 | 🞎 | 🞎 | 🞎 | 🞎 | 🞎 | 🞎 | 🞎 | 🞎 | 🞎 |
| Dentist |  |  | 🞎 | 🞎 | 🞎 | 🞎 | 🞎 | 🞎 | 🞎 | 🞎 | 🞎 | 🞎 |
| Dental Hygienist |  |  | 🞎 | 🞎 | 🞎 | 🞎 | 🞎 | 🞎 | 🞎 | 🞎 | 🞎 | 🞎 |
| Traditional Healers / Elders |  |  | 🞎 | 🞎 | 🞎 | 🞎 | 🞎 | 🞎 | 🞎 | 🞎 | 🞎 | 🞎 |
| Cultural Coordinators |  |  | 🞎 | 🞎 | 🞎 | 🞎 | 🞎 | 🞎 | 🞎 | 🞎 | 🞎 | 🞎 |
| Other, specify:  _____________________________________ |  |  | 🞎 | 🞎 | 🞎 | 🞎 | 🞎 | 🞎 | 🞎 | 🞎 | 🞎 | 🞎 |
| Other, specify:  _____________________________________ |  |  | 🞎 | 🞎 | 🞎 | 🞎 | 🞎 | 🞎 | 🞎 | 🞎 | 🞎 | 🞎 |
| Other, specify:  _____________________________________ |  |  | 🞎 | 🞎 | 🞎 | 🞎 | 🞎 | 🞎 | 🞎 | 🞎 | 🞎 | 🞎 |
| Other, specify:  _____________________________________ |  |  | 🞎 | 🞎 | 🞎 | 🞎 | 🞎 | 🞎 | 🞎 | 🞎 | 🞎 | 🞎 |

**SECTION 3: HEALTHCARE SERVICES AND DIABETES PROGRAMS**

1. Please check the appropriate box to indicate the **availability** (on-site, indirect, visiting, off-site) of **healthcare services, diabetes programs and supports** in your community. Certain services may be available through multiple sources – PLEASE MARK ALL THAT APPLY. Where applicable, please provide the estimated **frequency of visits** for visiting services/program and **distance** for off-site services/programs.

| **HEALTHCARE SERVICES AND DIABETES PROGRAMS** | **USUALLY AVAILABLE**  **ON-SITE** (i.e., live and work in the community) | **INDIRECTLY AVAILABLE** (i.e., through phone/internet, Telehealth) | **ONLY VISITING**  (e.g., fly-in, mobile truck) | | **ONLY AVAILABLE OFF-SITE**  (i.e., community members required to travel) | | | | | |
| --- | --- | --- | --- | --- | --- | --- | --- | --- | --- | --- |
|  |  |  | **Estimated # of visits per**  **(month OR year):** | | **Neighbouring Aboriginal Community** | **Non-Aboriginal Town or Urban Centre** | **Estimated Distance:** | | | |
|  |  |  | month | year |  |  | <20km | 20-40km | 40-80km | >80km |
| **CLINICAL SERVICES & PROGRAMS** | | | | | | | | | | |
| Dialysis treatment | 🞎 | 🞎 |  |  | 🞎 | 🞎 | 🞎 | 🞎 | 🞎 | 🞎 |
| Diabetes care and management (treatment and screening of complications, e.g., foot care) | 🞎 | 🞎 |  |  | 🞎 | 🞎 | 🞎 | 🞎 | 🞎 | 🞎 |
| Medication prescription | 🞎 | 🞎 |  |  | 🞎 | 🞎 | 🞎 | 🞎 | 🞎 | 🞎 |
| Laboratory services e.g. blood work, point of care testing, cultures | 🞎 | 🞎 |  |  | 🞎 | 🞎 | 🞎 | 🞎 | 🞎 | 🞎 |
| Vaccinations e.g. immunization clinics | 🞎 | 🞎 |  |  | 🞎 | 🞎 | 🞎 | 🞎 | 🞎 | 🞎 |
| Diabetes prevention program e.g. awareness and screening activities | 🞎 | 🞎 |  |  | 🞎 | 🞎 | 🞎 | 🞎 | 🞎 | 🞎 |
| **EDUCATION AND COUNSELLING** | | | | | | | | | | |
| Education & counselling for nutrition, healthy weight, physical activity, behaviour modification (e.g. smoking cessation) | 🞎 | 🞎 |  |  | 🞎 | 🞎 | 🞎 | 🞎 | 🞎 | 🞎 |
| Mental healthcare including psychosocial counselling | 🞎 | 🞎 |  |  | 🞎 | 🞎 | 🞎 | 🞎 | 🞎 | 🞎 |
| Substance abuse awareness activities; counseling for addictions | 🞎 | 🞎 |  |  | 🞎 | 🞎 | 🞎 | 🞎 | 🞎 | 🞎 |
| Other, specify:  _____________________________ | 🞎 | 🞎 |  |  | 🞎 | 🞎 | 🞎 | 🞎 | 🞎 | 🞎 |
| Other, specify:  _____________________________ | 🞎 | 🞎 |  |  | 🞎 | 🞎 | 🞎 | 🞎 | 🞎 | 🞎 |

| **2.** | Has your community implemented **innovative strategies** related to the following activities that have changed **diabetes care in your community**? PLEASE MARK ALL THAT APPLY. | | | | | | |  |
| --- | --- | --- | --- | --- | --- | --- | --- | --- |
| 🞎 | | Training for community healthcare providers/professionals | 🞎 | | Other (specify): |  |  |  |
| 🞎 | | Community health programs or interventions |  | |  | | | |
| 🞎 | | Health Research projects |  | |  | | | |
|  |  | |  |  | | | |  |

***Thank you very much for taking the time to complete this survey***
